# Cortical neuron calcium response to infrared neural stimulation depends on the spatiotemporal thermal gradient

**DOI:** 10.1364/BOE.603356

**Published:** 2026-07-29

**Authors:** Jacob Hardenburger, George Grow, Mona Gerges, Joel Bixler, Chad Oian, Bryan Millis, Christopher Valdez, E. Duco Jansen, Anita Mahadevan-Jansen

**Affiliations:** 1 Vanderbilt Biophotonics Center, Vanderbilt University, Nashville, TN, USA; 2 Department of Biomedical Engineering, Vanderbilt University, Nashville, TN, USA; 3 Air Force Research Laboratory, Bioeffects Division, JBSA Fort Sam Houston, TX, USA; 4 UT Health San Antonio, San Antonio, TX, USA; 5 Department of Neurological Surgery, Vanderbilt University Medical Center, Nashville, TN, USA

## Abstract

Infrared neural stimulation (INS) is a promising neuromodulation tool, yet its clinical translation is hindered by an incomplete understanding of how photothermal dynamics recruit specific physiological mechanisms. This study investigates how varying spatiotemporal thermal gradients influence calcium responses in primary rat cortical neurons by combining calcium imaging with numerical modeling and experimental validation. Laser dosimetry was performed by focusing a 1470 nm laser to a 15 µm diameter and varying the pulse energy for 0.5 ms, 5 ms, and 50 ms laser pulse durations. The thermal dynamics of the stimulus were simulated using a computational model, which was validated against thermal lensing experiments. Our results reveal a bimodal calcium response comprising high-frequency phasic spiking and low-frequency basal increases, which depend on the temporal thermal dynamics of the laser stimulus. This research provides a critical framework for designing spatially precise, effective, and safe INS technologies.

## 1. Introduction

The translation of infrared neural stimulation (INS) from a laboratory research tool to a clinically viable neurostimulation technology is currently limited by an incomplete understanding of thermal effects on complex physiological processes [1]. While INS has demonstrated superior spatial precision compared to electrical stimulation in several models [2–4], a cohesive theory explaining its underlying mechanisms remains elusive. Characterizing the parameter space in relation to physiological responses and, thereby, mechanisms would provide significant value to researchers seeking to advance the technology for clinical applications [5,6–9]. This is particularly important for central nervous system applications, where prior studies have shown diverse responses across multiple neural cell types [10–14].

Wells et al. were the first to investigate the biophysical mechanism underlying INS, concluding that it is photothermal in nature and primarily driven by thermal gradients [15]. Subsequent work confirmed that water is the primary chromophore and that infrared light converts into heat, altering membrane capacitance and generating a capacitive current [16]. Additional studies replicated these findings across several models [17–19], indicating that photothermal gradients fundamentally impact membranes through the rate of temperature change (d*T*/d*t*). Other research using spiral and vestibular ganglion neurons [20], dorsal root ganglion neurons [21], and Purkinje cells [22] suggests a dependence on absolute temperature change, implicating temperature-sensitive ion channels, particularly transient receptor potential vanilloid (TRPV) channels, in INS responses. Furthermore, intracellular calcium release has been observed in both neuronal and non-neuronal cells, hypothesized to originate from mitochondria [23] and the endoplasmic reticulum via IP_3_R [24]. However, it remains unclear whether this release is driven by thermal impulse, total thermal dose, or absolute temperature change. Additional studies have shown that INS can modulate neuronal excitability by altering synaptic kinetics [25–27]. Complicating matters, prior work from our lab demonstrated that altering laser exposure duration alone activates distinct molecular pathways in cortical astrocytes [28]. Collectively, these findings underscore the uncertainty surrounding how experimental parameters recruit multiple mechanisms.

Within the cortex, the relative influence of optocapacitive currents versus other potential INS effects remains unclear, as highlighted by the diversity of responses reported by Fu et al. [12]. The parameter space and its relation to mechanisms in the brain have been relatively unexplored since seminal work in thalamocortical brain slices [29]. Since then, most in vivo brain applications have employed pulse trains consisting of 100 individual, 250 µs pulses repeated at 200 Hz over a total duration of 500 ms [14,28,30,31], generating a slow thermal gradient that differs fundamentally from the initial biophysical mechanism proposed by Wells.

Analytical approaches for INS have proposed that thermally confined laser pulses are the most efficient means of delivering laser light into tissue [32]. This is supported by experimental findings in the peripheral nervous system [33]. Thermal confinement occurs when the laser pulse duration is shorter than the thermal relaxation time, analytically defined as:

(1) $$\tau_{\text{Therm}}=\frac{\ell^{2}}{4\alpha }$$

where *ℓ* is the characteristic length determined by the irradiation beam geometry and optical penetration depth, and α is the thermal diffusivity of the irradiated medium [34]. However, given the non-thermally confined laser heating durations typically used *in vivo* in the brain, that is $\tau_{\text{pulse}}>>\tau_{\text{Therm}}$ , latent heat diffusion may activate distinct mechanisms. To date, little work has evaluated how controlled heating duration affects calcium signaling in cortical neurons, a critical signaling ion involved in numerous cellular processes and an indicator of electrical activity [35].

The transition from thermally confined to non-confined laser pulses requires a shift in the approach to laser dosimetry. Irradiation geometry fundamentally influences both spatial and temporal thermal dynamics, as emphasized by our previous work, which showed the importance of considering the laser delivery method and beam profile when determining optical dosages [36]. For non-thermally confined pulses, conventional dosage metrics such as radiant exposure or power become less informative because of thermal diffusion. Instead, the temperature dynamics at the cells become the most meaningful dosage metric, but determining these dynamics can be challenging. Direct measurement of the spatiotemporal thermal gradient is challenging for small spot sizes and is influenced by surrounding material properties [37]. Numerical modeling of photothermal interactions offers an alternative to capture both spatial and temporal dynamics with high fidelity [38]. Accurately capturing the thermal dynamics is essential for understanding the physiological mechanisms underlying INS.

The goal of this study is to determine how varying spatiotemporal thermal gradients affect calcium responses and the mechanisms recruited in cortical neurons. We develop a numerical model to calibrate optical doses to local temperature increases at the cellular level and validate the predicted thermal dynamics experimentally using thermal lensing. Using this approach, we demonstrate that calcium responses are bimodal, dependent on heating duration, and are fundamentally influenced by the spatial extent of the thermal gradient. In the discussion, we explore potential mechanisms underlying these observations and their relationship to spatiotemporal thermal dynamics.

## 2. Methods

### 2.1. Primary neuron culture

Cortices from E18 Sprague Dawley rats (Transnetyx Tissue, KTSDECX) were dissociated and cultured according to Brewer et al. [39]. One day before plating, 14 mm glass-bottom imaging wells in 35 mm Petri dishes (P35G-0-14-C, MatTek) were coated in a sterile hood with Poly-D-Lysine (A3890401, ThermoFisher) and incubated for 12–15 hours at room temperature. On the day of plating, dishes were washed once with sterile water and allowed to dry for 2 hours. The cortical tissue was incubated for 10 minutes in papain at 2 mg/mL in calcium-free Hibernate E media. The tissue was then triturated with a fire-polished pipette to disperse the cells, and then passed through a 100 μm strainer to remove undissociated tissue. The cells were pelleted by centrifugation at 1100 rpm for 1 minute. Following centrifugation, the supernatant was discarded, and the pellet was resuspended in growth media consisting of 97.75% Neurobasal Plus, 2% B-27 Plus, and 0.25% GlutaMAX. After counting with a hemocytometer, approximately 50,000–60,000 neurons were plated in 400 μL of media per imaging well, corresponding to a final density of ∼325–390 cells/mm^2^. Dishes were placed in an incubator at 37 °C with 5% CO_2_, and an additional 1.5 mL of growth media was added 3 hours post-plating. Neurons were maintained by partial media changes of 1 mL every 3 days and were used for experiments between 14 and 28 days in vitro.

### 2.2. Infrared neural stimulation

**Fig. 1.**
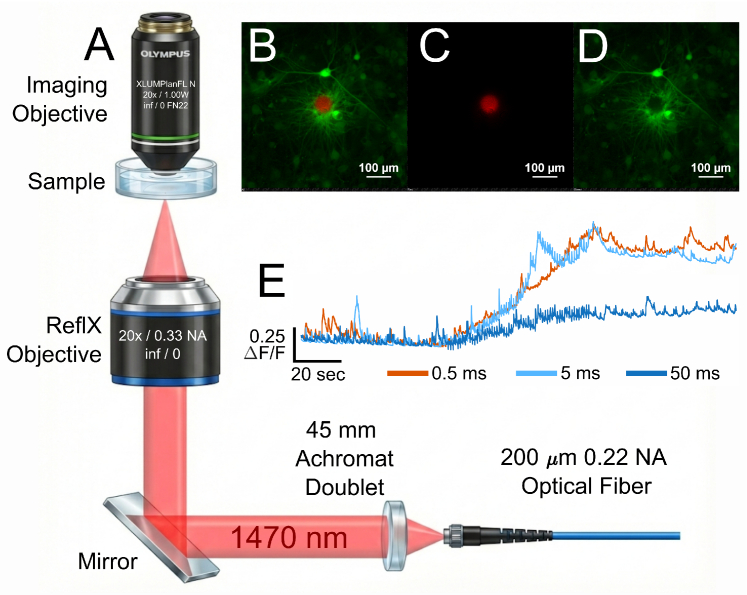
A) Experimental setup for INS experiments using a reflective objective to focus the laser stimulation light to target single cells. B) Composite image of red laser pilot light (C) and cortical neurons stained with Calbryte-520 (D) following application of a laser pulse to ablate a neuron under the pilot light, highlighting the spatial confinement of the laser energy density. E) Representative traces of neuronal calcium responses to the stimulation protocol for 0.5 ms, 5 ms, and 50 ms pulse durations.

Two laser delivery configurations were used to evaluate the effects of temporal and spatial thermal gradients on cortical neurons. Infrared laser irradiation was delivered using a custom 20 W, 1470 nm diode laser (Innovative Photonics Solutions, Monmouth Junction, NJ) coupled into a 200 µm or 400 µm optical fiber. In the first configuration, laser light was collimated from a 200 µm optical fiber using a 45 mm focal length achromatic doublet. The collimated beam was directed into a 0.33 NA reflective objective (39-142, Edmund Optics, Barrington, NJ) positioned beneath the Petri dish containing cortical neurons, parfocal with the imaging objective, to focus the stimulation light onto a single cell (Fig. 1(A)). The irradiation spot size was adjusted by translating the optical fiber toward the coupling lens while monitoring the red pilot beam at the imaging plane. The final spot diameter at the sample plane was 15 µm, yielding a 95 µs thermal time constant (Fig. 1(B), (C)). In this configuration, three pulse durations: 0.5 ms, 5 ms, and 50 ms, were tested at varying laser power levels to assess the effect of heating duration on neuronal calcium release. The second laser delivery configuration used a bare 400 µm optical fiber positioned 200 µm below and orthogonal to the bottom of the Petri dish using a micromanipulator, resulting in a 460 µm spot diameter (Fig. S1A) and a 89.4 ms thermal time constant [36]. Laser pulses were delivered at 1 Hz for one minute, resulting in 60 pulses in total (Fig. 1(E)). The dosages applied using the 15 µm diameter spot for each pulse width are summarized in Table 1. The optical setup was calibrated before each experiment, and the laser power at the sample plane was measured using a power sensor (S415C, Thorlabs, Newton, NJ).

**Table 1.** Laser exposure dosages presented for each pulse duration for the 15 µm spot size.

Pulse Duration [ms]	Number of Cells (n)	Number of Dishes (N)	Power [mW]	Radiant Exposure [J cm^-2^]	Maximum Temperature Change [°C]
0.5	65	20	79.6-116.8	22.5-33.0	28.0-41.1
5	55	11	28.4-57.9	80.4-163.9	18.7-38.2
50	59	13	16.1-39.0	456.5-1103.5	14.4-34.9

### 2.3. Calcium imaging

Calcium imaging was conducted to assess neuronal responses to INS. Prior to imaging, cultured neurons were incubated with 1.5 µM Calbryte-520 AM (AAT Bioquest) for 30 minutes at 37 °C in a humidified incubator with 5% CO_2_. After incubation, cells were rinsed with electrophysiology solution containing: 133 mM NaCl, 4 mM KCl, 1 mM MgCl_2_, 2 mM CaCl_2_, 1.5 mM TES sodium salt, and 11 mM D-glucose, to remove excess dye. Imaging was performed under an upright epifluorescence widefield microscope [36]. This configuration provides a resolution of 240 nm and an effective magnification of 44x. Calbryte-520 fluorescence was excited using the 488 nm line from a multi-band LED light engine (Spectra III, Lumencor, Beaverton, Oregon). Excitation and emission light were separated using a penta-band dichroic mirror (FF409/493/573/652/759-Di01-25 × 36, Semrock, Rochester, NY), and emission was filtered using a bandpass filter (FF01-538/685-25, Semrock) before detection with a back-thinned sCMOS camera (FusionBT, Hamamatsu Photonics) (Fig. 1(B), (D)). Imaging was performed at 20 Hz for a total of 3 minutes: the first minute captured baseline neuronal activity, laser stimulation was applied at 1 Hz during the second minute, and the final minute monitored post-stimulation calcium responses to assess recovery and sustained signaling (Fig. 1(E)). All imaging was performed at 37 °C using a heated-stage incubator (Tokai Hit) to maintain cell viability during experimentation. Calbryte-520 fluorescence was measured within a 15 µm diameter circular region of interest (ROI) covering the neuronal soma (Fig. 1(C)).

The source of the calcium response was investigated by stimulating cells under calcium-free (Ca_(-)_) conditions, in which the calcium in the electrophysiology solution was replaced with 2 mM Mg. A negative control group (Control_(-)_) was used to help contextualize the results from the Ca_(-)_ conditions. Cells in this group were loaded with BAPTA-AM (ThermoFisher, B1205) in addition to Calbryte-520 to chelate intracellular calcium before imaging in Ca_(-)_ media. The positive control group (Control_(+)_) consisted of cells exposed in electrophysiology solution.

### 2.4. Calcium analysis

Analysis of calcium fluorescence intensity data was performed using a custom MATLAB script (Fig. S2). The fluorescence traces were processed to separate high-frequency calcium activity related to firing and phasic neuronal modulation from tonic baseline increases, and the resulting data were quantified across the three experimental phases. For each measurement, fluorescence intensity was baseline-normalized (ΔF/F_0_). The signals were decomposed into DC and AC components. The DC component was estimated by applying a moving median filter over a 5-second window to remove high-frequency activity. The AC component was obtained by subtracting the DC component from the baseline-normalized intensity. Thermal lensing artifacts in the data appeared as negative deflections in the fluorescence signal [36,40]. Therefore, negative values in the AC component were set to zero to remove these artifacts. Calcium events were identified using a derivative-based detection method applied to the AC component of the signal. A dynamic noise threshold was calculated for each trace based on the variability of nonzero signal data, defined as the median plus the mean absolute deviation (MAD). Regions in the signal where spiking events were present were identified when three criteria were satisfied: (1) the AC signal amplitude exceeded the noise threshold, (2) the signal derivative was classified as a statistical outlier using a Grubbs’ test, and (3) the derivative indicated a positive rising edge. Local maxima were identified within these regions of the AC signal. The number of spiking events and the mean event height were computed per neuron for each experimental phase to quantify the AC signal. The DC signal was quantified by computing the area under the curve (AUC, ΣΔF/F_0_) during each experimental phase. Spiking activity was normalized to baseline by subtracting the number of events detected before stimulation from the number of events detected during stimulation. Neurons were classified as unreactive, reactive, or damaged based on their change in spiking activity and AUC during stimulation relative to a Sham group of unstimulated neurons and a damage group based on a damage assay. Reactive neurons exhibited either normalized spiking activity or a baseline rise exceeding the average plus three standard deviations observed in the Sham group for each metric.

### 2.5. Damage assay

A damage assay was performed to correlate neuronal calcium responses with membrane damage. Neurons were stained with Calbryte-520 following the calcium imaging protocol described above. Prior to imaging, 1 µM of propidium iodide (PI) was added to the dish to mark non-membrane-bound DNA. The acquisition protocol for the damage assay was automated using a custom JOB built in NIS-Elements, following the protocol in [36]. For each experiment, an image of the pre-stimulation PI signal was captured to measure baseline signal intensity. Propidium iodide was excited at 550 nm, and emission was detected after filtering with a 624/40 nm bandpass filter. Calcium imaging was performed according to the protocol described above. Thermal dosages were selected based on their ability to elicit a baseline rise in calcium near the upper end of the range in Table 1. Image acquisition was then paused for 30 minutes to allow PI to bind to any exposed DNA before capturing an image of the post-exposure PI signal. The PI intensity was measured within the same ROI as the calcium fluorescence intensity. The change in PI intensity was baseline-normalized using the pre-stimulation PI signal. The calcium increase during stimulation was correlated with the increase in PI intensity to predict whether a cell was damaged. The change in PI intensity was measured against a Sham group of unstimulated cells. Cells showing a change in PI intensity greater than the mean plus three standard deviations of the Sham group were considered damaged. Cells below this threshold were used to set the damage cutoff based on their AUC during stimulation, using the mean plus one standard deviation to be conservative and avoid including damaged cells.

### 2.6. Optical-thermal model

The quantification of spatiotemporal thermal gradients involves coupling the radiative transport equation with the heat conduction equation, which describes the propagation, dispersion, and absorption of light within a medium. Monte Carlo is commonly used to estimate light distributions by propagating individual photons according to probability distributions specified by the optical properties of the domain. The absorption of light results in three-dimensional energy deposition, which is converted into heat, depending on the thermal properties. Heat conduction through the material is subsequently determined by Pennes’ bioheat equation. The Scalable Effects Simulation Environment (SESE) uses Monte Carlo and numerical methods to solve this process within an inhomogeneous structure [38].

**Fig. 2.**
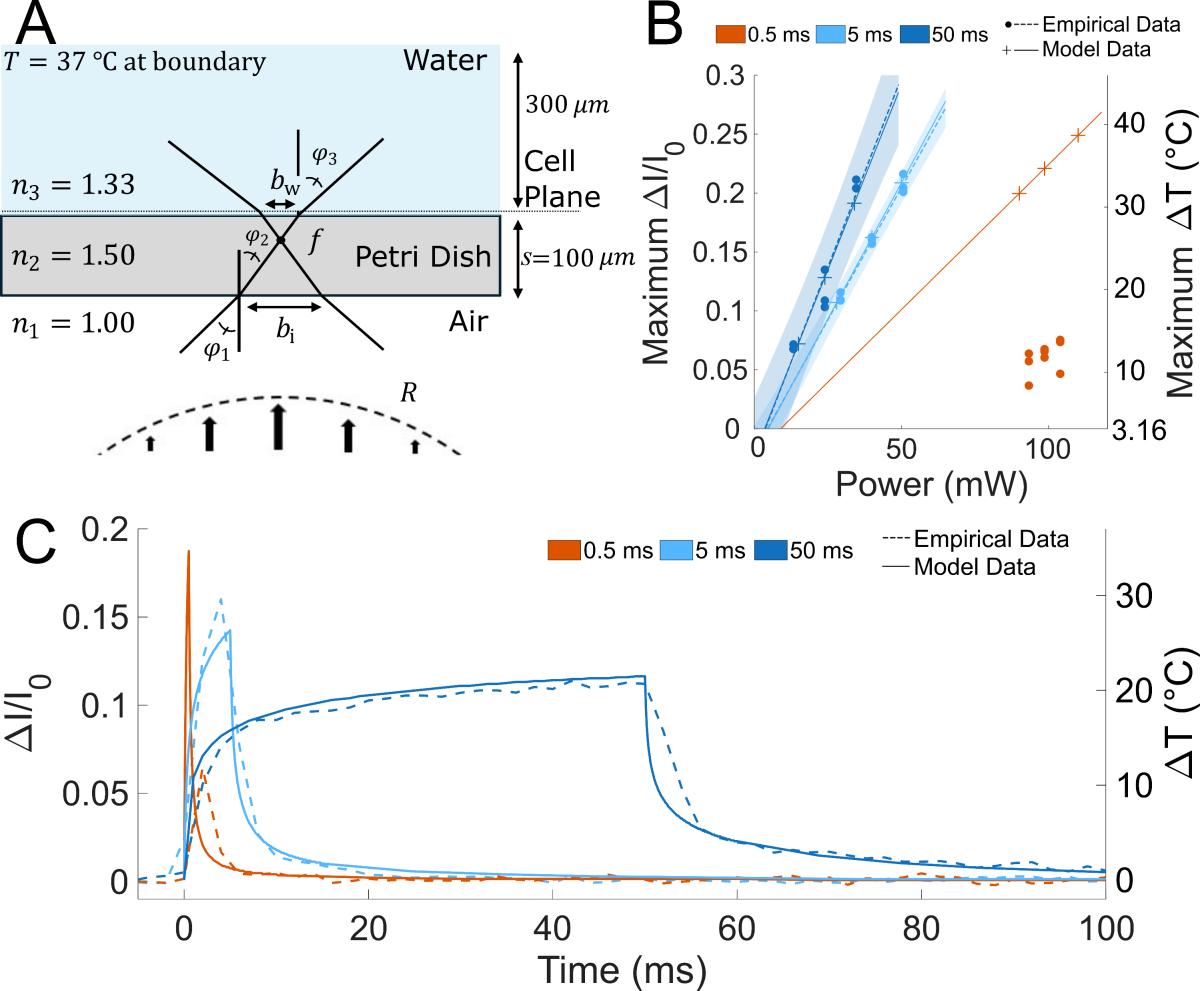
Thermal model results and experimental validation. A) Geometry of the thermal model used to apply INS through focusing optics. B) Comparison of empirical and modeled data: the left y-axis shows the maximum change in pilot light intensity during irradiation (empirical), while the right y-axis shows temperature dependence on power (model). Empirical data (●) are plotted with dashed lines and 95% confidence intervals represented by the shaded area; modeled data (+) are plotted with solid lines. The lower bound of the right y-axis is the limit of detection for the thermal lensing experiment, where no change in intensity can be measured. (n = 3 trials per pulse duration and power) C) Representative thermal profiles from thermal lensing experiment (dashed traces, left y-axis) and numerical simulations (solid lines, right y-axis) for a 98.6 mW, 0.5 ms pulse; 40.0 mW, 5 ms pulse, and 24.0 mW, 50 ms pulse.

A (0.8 × 0.8 × 0.5) mm domain with a 2 µm resolution was initialized with symmetry imposed across the x-z and x-y planes to reduce computation time. Dirichlet boundary conditions were imposed along the periphery of the model to allow heat efflux. The optical and thermal properties of water and glass were used to populate the two inhomogeneous domains in the model (Table 2). To model the stimulation geometry (Fig. 2(A)), a non-zero beam waist at the focal plane was approximated by placing the simulated focal point just below the cellular plane to achieve the desired beam waist $\left(b_{w}\right)$ and divergence angle $\left(\phi_{3}\right)$ at the cell plane. Snell’s law was used to determine the divergence angle of light through the slide $\left(\phi_{2}\right)$ with a refractive index (*n_2_* = 1.50). The diameter of the incident beam $\left(b_{i}\right)$ on the underside of the glass slide can then be determined using the thickness of the slide (*s*) (Eq. (2)).

(2) $$b_{i}=\text{tan}(\phi_{2})s-b_{w}$$

(3) $$f=s-\text{cot}(\phi_{2})b$$

(4) $$R=2f(n_{2}-1)$$

**Table 2.** Material properties used in the thermal model**.**

λ = 1470 [nm]	Refractive Index (n)	Optical Absorption (μ_a_) [m^-1^]	Density (ρ) [kg/m^3^]	Specific Heat (c) [J/kgK]	Thermal Conductivity (k) [W/mK]
Electrophysiology Media (H_2_O)	1.33	2510	997	4182	0.7
Petri Dish (B_2_O_3_)	1.50	0	2200	830	1.1

The photons are launched according to the curvature radius (*R)* and focal length (*f)* specified by the thin-lens approximation of the lens-maker equation (Eq. (3), (4)). All simulations were carried out on a workstation equipped with 3rd Gen AMD RyzenTM ThreadripperTM processors (32 cores at 3.7 GHz), a Samsung 970 EVO Plus 1 TB NVMeTM solid-state drive, and 128 GB of RAM running an Ubuntu Linux desktop environment using SESE source code branched from version 2.6.0.

### 2.7. Thermal lensing experiments

Thermal lensing was used as a surrogate for heating to validate the numerical thermal model. The experimental setup was configured to match the calcium imaging protocol. An empty imaging dish, devoid of neurons, was filled with electrophysiology solution and placed under the microscope. For both laser delivery configurations, the red pilot light of the laser was turned on and used as the light source to visualize the thermal lensing effect [36]. The pilot light was imaged for 2 s at 500 Hz, and the laser was triggered manually during imaging. The pilot light intensity was measured at the center of the imaging field using a 15 µm diameter ROI concentric with the pilot light. The pilot light intensity was baseline-normalized, and the thermal relaxation time, defined as the time for the laser pilot light to relax to 1/e of its peak, was extracted from the signal. The maximal change in intensity was correlated with power and compared with the maximal rise in temperature predicted by the thermal model.

## 3. Results

### 3.1. Thermal model and experimental validation

The infrared-induced thermal gradient was modeled using SESE for each of the three pulse durations at the tested power levels for the 15 µm spot size (Table 1). For all pulse durations, the simulated temperature profile scaled linearly with input power (Fig. 2(B)). To validate the model, thermal lensing experiments were performed to monitor changes in the laser pilot light during stimulation ( Visualization 1 and Visualization 2). The pilot light intensity changes linearly with laser power for the 5 ms and 50 ms pulse durations (Fig. 2B; R^2^ = 0.9858 and 0.9390, respectively). Linear fits of the numerical data for the 5 ms and 50 ms pulses fall squarely within the 95% confidence interval of the regression line of the empirical data. The detection limit for thermal lensing was approximately 3.16 °C, as indicated by the comparison of empirical and numerical data in Fig. 2B. The shapes of the empirical and numerical profiles during irradiation were similar for the 5 ms and 50 ms pulse durations (Fig. 2C; RMSE = 0.077 and 0.070, respectively). While the 0.5 ms pulse could not be empirically validated due to sampling-rate constraints, the strong agreement for longer pulses, where the Nyquist criterion was satisfied (f_Nyquist_ = 400 Hz and 40 Hz for 5 ms and 50 ms pulse durations), supports the model accuracy in capturing temporal thermal dynamics.

### 3.2. Cortical neuron calcium pulse duration dependence

**Fig. 3.**
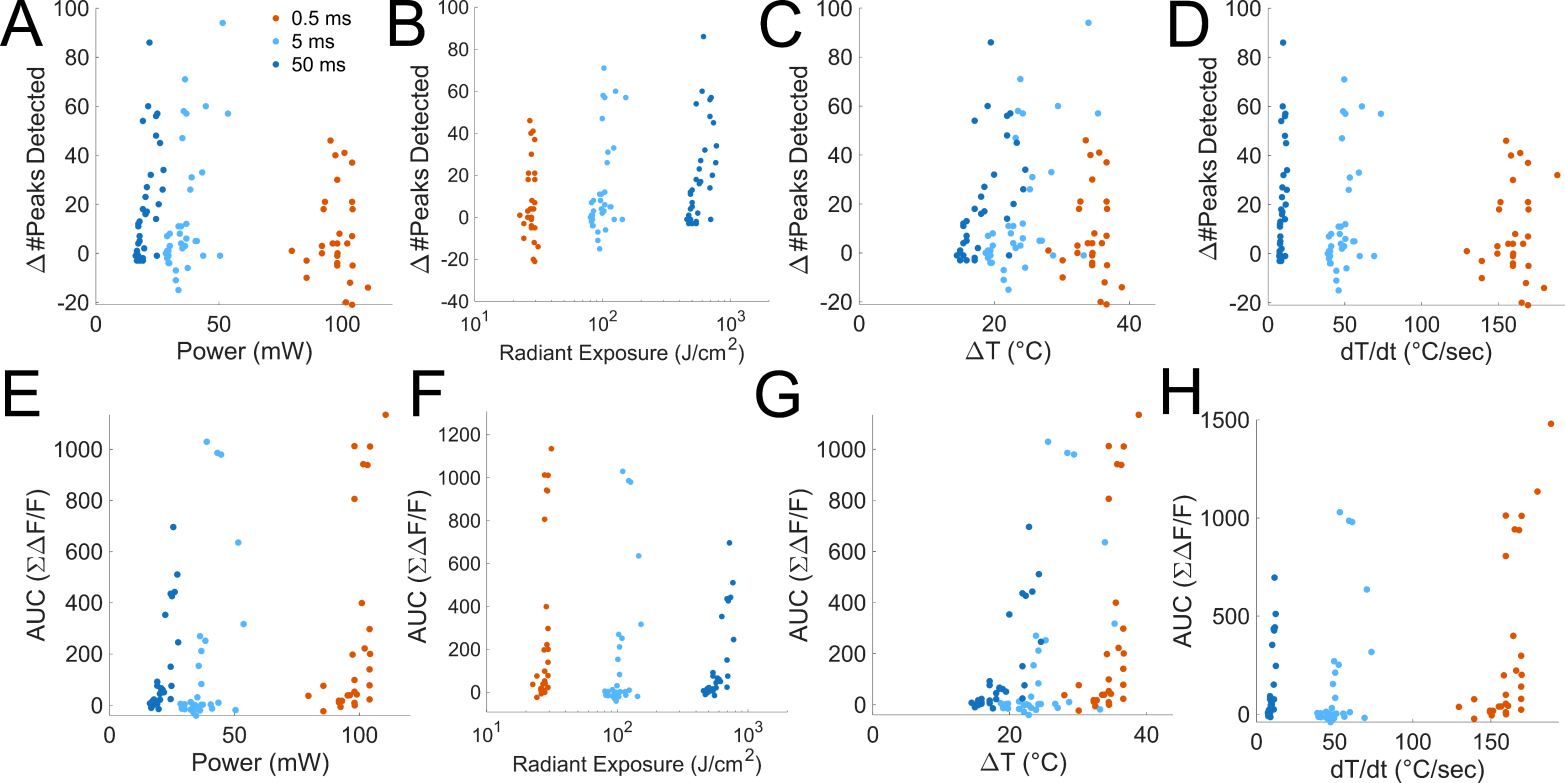
Dose-Response plots. A-D) Correlation between power (A), radiant exposure (B), maximum temperature rise (C), and maximum rate of temperature increase (D) to baseline-normalized spiking activity. E-H) Correlation between power (E), radiant exposure (F), maximum temperature rise (G), and maximum rate of temperature increase (H) to basal calcium levels observed during stimulation. (n = 29, 38, and 33 cells for the 0.5 ms, 5 ms, and 50 ms pulse durations, respectively)

Neuronal responses showed a clear temperature-time dependence as observed in Fig. 3. The power required to elicit a response was inversely related to pulse width: 98.6 ± 6.78 mW, 39.91 ± 6.47 mW, and 22.1 ± 3.23 mW, for 0.5 ms, 5 ms, and 50 ms pulses, respectively (Fig. 3(A), (E)). The order-of-magnitude separation in pulse duration produces a roughly fivefold difference in radiant exposure: 27.9 ± 1.9 J/cm^2^, 113 ± 18.3 J/cm^2^, and 624 ± 91.4 J/cm^2^ for 0.5 ms, 5 ms, and 50 ms pulses, respectively (Fig. 3(B), (F)). Despite requiring the highest radiant exposure, the 50 ms pulse elicited a calcium response at the smallest temperature increase (19.8 ± 2.89 °C, reaching ∼56.8 °C absolute). The 5 ms pulse required a 26.3 ± 4.27 °C increase, while the 0.5 ms pulse required the largest increase (34.69 ± 2.38 °C) (Fig. 3(C), (G)). Similarly, the 0.5 ms pulse duration yielded the largest rate of temperature change at 161.7 ± 12.2 °C/sec, whereas the rate of change was lower for the 5 and 50 ms pulse durations at 54.7 ± 8.9 °C/sec and 9.9 ± 1.5 °C/sec (Fig. 3(D), (H)). Correlation analysis revealed moderate relationships between dosage and spiking activity for 5 ms and 50 ms pulses (Pearson’s r = 0.5596; 0.5538; p = 0.0003; 0.0008), but no significant correlation for 0.5 ms pulses (Pearson’s r = –0.005, p = 0.9814). All pulse durations showed moderate correlations between dosage and basal calcium increases during stimulation (Pearson’s r = 0.4972; 0.5136; 0.7686; p = 0.007; 0.001; <0.001 for 0.5 ms, 5 ms, and 50 ms, respectively) (Fig. 3(E)–(H)).

**Fig. 4.**
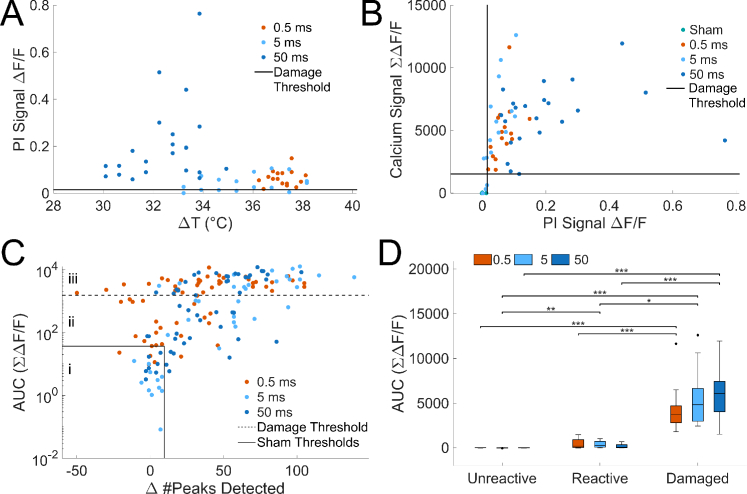
A) Relationship between maximum temperature rise and propidium iodide (PI) fluorescence; the damage threshold determined by the Sham group is indicated as a solid line. B) Correlation between basal calcium levels during stimulation and PI expression, with damage thresholds for each metric indicated by solid lines (n = 8, 16, 16, and 22 cells for the Sham, 0.5 ms, 5 ms, and 50 ms groups, respectively). C) Scatter plot illustrating the relationship between the baseline normalized spiking activity and the AUC during stimulation, with data color-coded by laser pulse duration. Zones showing reaction phenotype are labeled as i) unreactive cells, ii) reactive cells, and iii) damaged cells. D) Comparison between AUC observed across pulse duration and response phenotypes (n(i) = 7, 25, 11 cells; n(ii) = 22, 13, 22 cells; n(iii) = 36, 17, 26 cells for the 0.5 ms, 5 ms, and 50 ms pulse durations for each phenotype, respectively). *p < 0.05; **p < 0.01; ***p < 0.005.

Cell damage occurred for each pulse duration tested and exhibits a temperature-time dependence (Fig. 4). The maximal increase in PI fluorescence of the Sham is ∼1.5%, indicating that cells above this level were likely damaged (Fig. 4A, S3). The 50 ms pulse required the lowest power to elicit PI uptake (36.2 ± 1.5 mW; ∼32.4 ± 1.37 °C rise), while 5 ms and 0.5 ms pulses required 54.2 ± 2.48 mW and 105.7 ± 1.51 mW, corresponding to 36.0 ± 1.64 °C and 37.2 ± 0.53 °C rises, respectively. The PI fluorescence changes show a moderate correlation with basal calcium increases during stimulation (Spearman’s ρ = 0.68, p < 0.0001, Fig. 4(B)). Neurons displaying an AUC less than 36.7 during stimulation or less than 9 additional spikes compared to the pre-stimulation period react no differently than the unstimulated Sham group (Fig. 4(C)). The damage group shows a significant increase in basal intracellular calcium during stimulation compared with both the unreactive and reactive groups (Fig. 4(D)). Reactive cells exposed to 0.5 ms and 50 ms pulses show no statistically significant difference in basal calcium levels during stimulation compared to the unreactive group. However, cells in the 5 ms group exhibited a higher calcium level than those in the unreactive group. There was no difference in AUC during stimulation between pulse durations within the reactive group. Both reactive and damaged cells retained elevated intracellular calcium levels following stimulation. There is a strong correlation between the increase in basal calcium during stimulation and the elevated level following stimulation (Pearson’s r = 0.9036, 0.8820, 0.9346 for 0.5 ms, 5 ms, and 50 ms, respectively; p < 0.0001 for all pulse durations).

**Fig. 5.**
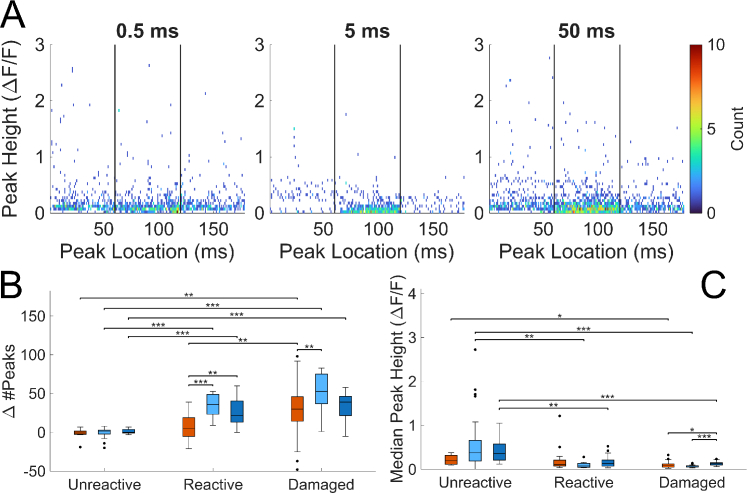
A) 2-dimensional histograms showing temporal distribution of spiking activity against spike height in the Reactive group; x-bin width = 1 second; y-bin width = 0.05 ΔF/F. Vertical black lines indicate the beginning and end of 1 Hz laser stimulation. The color scale represents the density count of overlapping data points. B) Comparison of changes in peak count across response categories and pulse durations. C) Comparison of median peak height across response categories and pulse durations (n = 7, 25, 11 cells; n = 22, 13, 22 cells; n = 36, 17, 26 cells for the 0.5 ms, 5 ms, and 50 ms pulse durations for the Unreactive, Reactive, and Damaged phenotypes, respectively). *p < 0.05; **p < 0.01; ***p < 0.005.

Neurons in the reactive group exposed to 5 ms and 50 ms pulses show elevated spiking activity during stimulation, which returned to baseline after the laser was turned off (Kruskal–Wallis test with Bonferroni correction, p < 0.0001 between experimental phases for both pulse widths; Fig. 5A, Visualization 3 and Visualization 4). In contrast, neurons in the reactive group exposed to 0.5 ms pulses did not show elevated spiking activity during stimulation ( Visualization 5). Overall, 5 ms and 50 ms pulses were more effective than 0.5 ms pulses at eliciting spiking activity (Fig. 5(B)). Spiking activity in the damage group was significantly elevated for all pulse widths. The mean peak height per neuron was significantly lower than that of the unreactive group for the 5 ms and 50 ms pulse durations (Fig. 5(C)). However, spiking activity levels within the reactive group across the experimental phases did not differ significantly.

**Fig. 6.**
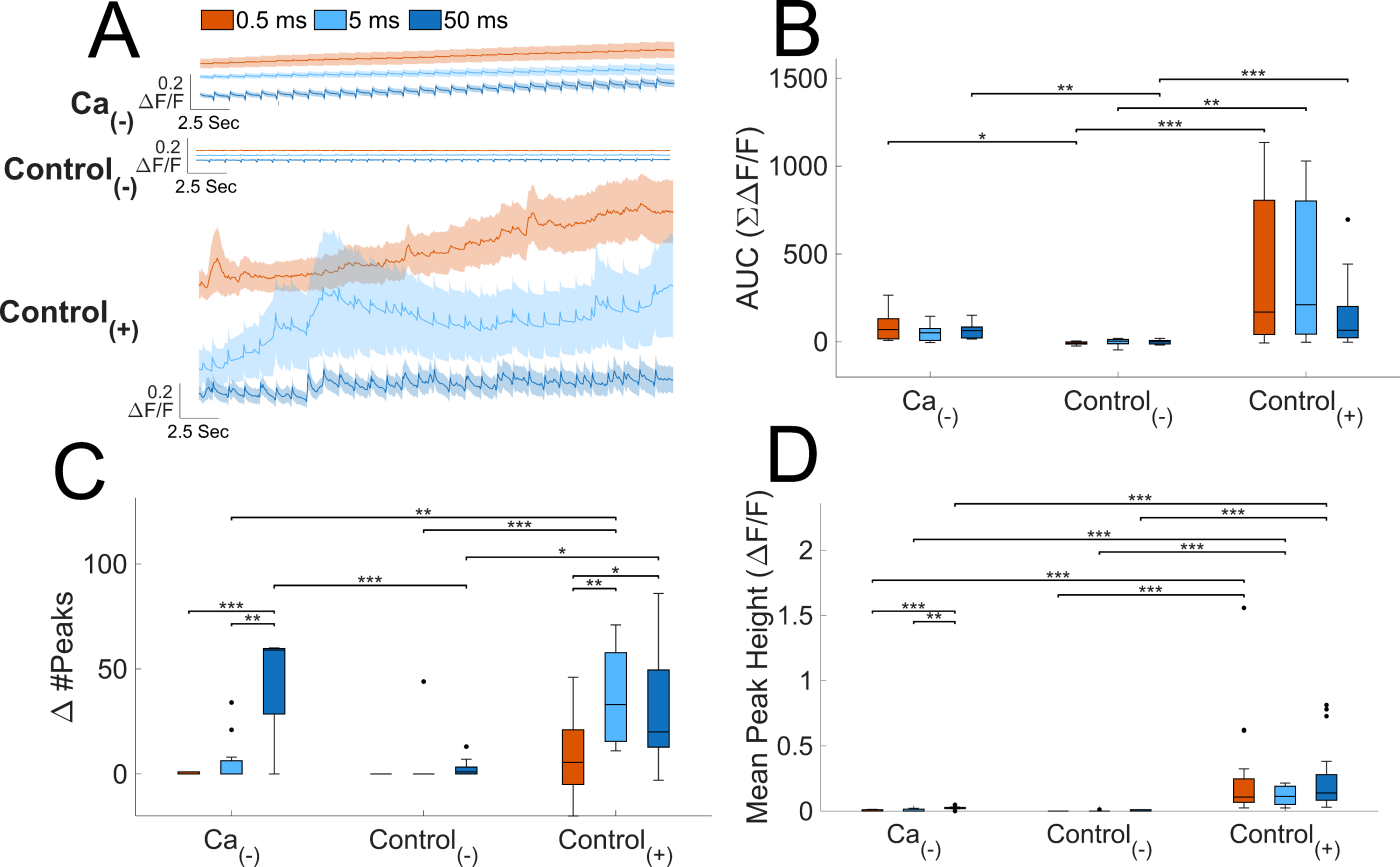
A) Calcium response during stimulation for each condition: calcium-free media (Ca_-_), negative control (BAPTA-AM + Ca_-_), and positive control, shown as group means with standard error shading, color-coded by pulse duration. B) AUC comparison across experimental conditions and pulse durations. C) Baseline-adjusted spiking activity compared across conditions and pulse durations. D) Mean peak height comparison across conditions and pulse durations (n = 10, 11, 11 cells; n = 9, 10, 9 cells; n = 22, 11, 21 cells for the 0.5 ms, 5 ms, and 50 ms pulse durations under the Ca_(-)_, Control_(-)_, and Control_(+)_ conditions, respectively). *p < 0.05; **p < 0.01; ***p < 0.005.

Removing calcium from the media reduced spiking activity but did not prevent basal intracellular calcium increases. Chelating intracellular calcium with BAPTA-AM in addition to calcium-free media abolished both spiking activity and basal calcium elevation (Fig. 6(A)). Changes in basal intracellular calcium levels showed no statistical difference between the calcium-free group and the positive control group (Fig. 6B; p > 0.05, Kruskal–Wallis test with Bonferroni correction). The 0.5 ms and 50 ms pulse durations in the Ca_(−)_ condition show elevated intracellular calcium levels compared to the negative control group. The 50 ms pulse duration elicits greater spiking activity in the Ca_(−)_ condition than either the 5 ms or 0.5 ms durations (Fig. 6C; p < 0.01, Kruskal–Wallis test with Bonferroni correction). Additionally, the 50 ms pulse was the only pulse duration that showed significantly greater spiking activity under Ca_(−)_ conditions compared to the negative control group. The 5 ms pulse duration was the only condition that displayed significantly different spiking activity compared to the positive control group. The magnitude of spikes under Ca_(−)_ conditions was not statistically different from the negative control group, with the 50 ms pulse showing the largest increase at 2.45 ± 0.24% (mean ± SD). These spikes were significantly smaller than those observed in the positive control group (Fig. 6(D)).

### 3.3. Influence of spatiotemporal gradient on neuronal reactions

**Fig. 7.**
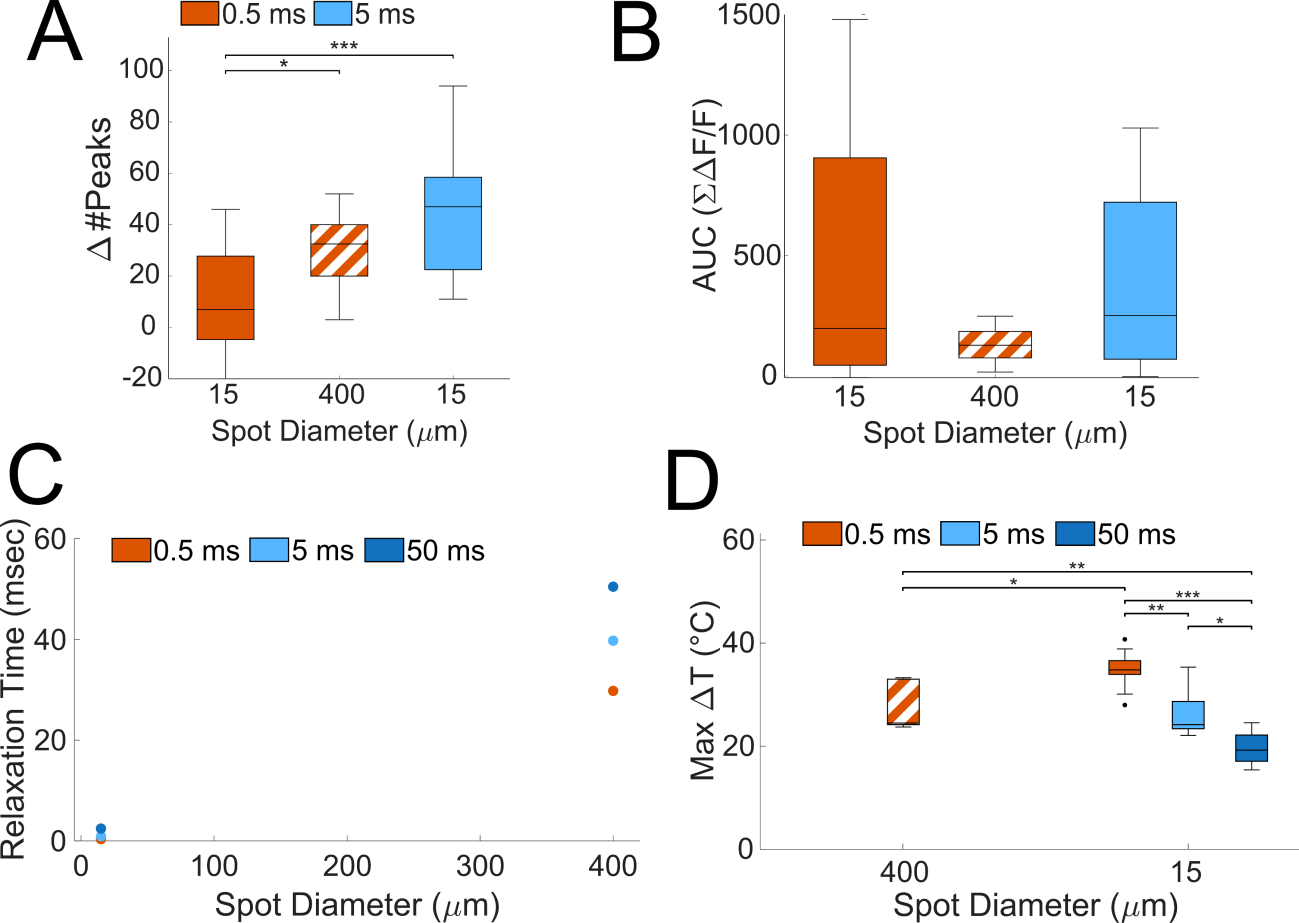
A) Baseline-adjusted spiking activity compared across a 15 µm diameter spot (0.5 ms and 5 ms pulses) and a 400 µm diameter spot (0.5 ms pulse). B) AUC during stimulation for the same groups shown in panel A. C) Thermal relaxation dependence on laser spot size provided by the numerical model. D) Maximum temperature rise predicted by the numerical model (n = 23, 13, and 22 cells for the 0.5 ms, 5 ms, and 50 ms 15 µm diameter spot; n = 15 cells for the 400 µm diameter spot, Kruskal-Wallis *p < 0.05; **p < 0.01; ***p < 0.005).

The effect of the spatiotemporal thermal gradient on confinement time becomes critical during stimulation. Applying a 0.5 ms pulse with a 400 µm spot size resulted in spiking activity comparable to that elicited by a 5 ms pulse applied with a 15 µm spot (Fig. 7(A)). Additionally, the 400 µm spot produced significantly more spiking activity than the 15 µm spot when pulse duration was held constant (p = 0.032, Kruskal–Wallis test with Bonferroni correction). There was no statistical difference in basal calcium release between the two spot sizes when pulse duration was held constant (Fig. 7(B)). Using the numerical model, the thermal relaxation time for a 15 µm spot was calculated to be 340 µs, 909 µs, and 2.44 ms for pulse durations of 0.5 ms, 5 ms, and 50 ms, respectively. Increasing the spot diameter by delivering irradiation through a 0.22 NA, 400 µm optical fiber resulted in much longer thermal relaxation times: 29.8 ms, 39.7 ms, and 50.5 ms for the 0.5 ms, 5 ms, and 50 ms pulses, respectively (Fig. 7(C)). While the maximum temperature rise of the 0.5 ms pulse out of the 400 µm spot was the most similar to the 5 ms pulse duration of the 15 µm spot diameter at 24.5 ± 4.2 vs 24.2 ± 3.4 °C (median ± MAD), respectively (Fig. 7(D)).

## 4. Discussion

In this study, we evaluate how spatiotemporal thermal gradients influence calcium activity in cortical neurons. Our findings show that neuronal responses depend on both the magnitude and duration of the thermal gradient. Specifically, the data indicate that a thermal gradient must persist for at least 5 ms to elicit phasic spiking calcium activity. However, all tested pulse durations produced low-frequency basal increases in intracellular calcium, suggesting that at least two distinct mechanisms contribute to the observed calcium response observed in neurons. Removing extracellular calcium reduced spiking activity but did not diminish the basal calcium increase, indicating that intracellular calcium stores are partially responsible for the basal signal (Fig. 6). Additionally, we demonstrate that increasing the laser spot size, resulting in thermal confinement, significantly affects neuronal responses.

We used numerical modeling to assess the spatiotemporal thermal dynamics of the stimulus. This approach offers distinct advantages over traditional measurement techniques previously employed to quantify laser-induced heating in neurons, such as electrode resistance using patch-clamp methods [41]. Thermal modeling enables an accurate assessment of thermal dynamics influenced by material properties, as noted by Thompson et al. [37], which is particularly important for temporal thermal dynamics near a heat sink. Introducing an electrode via a patch creates a heat sink within the cell, altering temporal thermal dynamics and potentially confounding mechanistic interpretations, as indicated by our data. Modeling eliminates the need for a patch that could be compromised by thermal gradients, reducing the risk of misleading conclusions about physiological mechanisms.

The importance of multiphysics computational modeling for INS studies, particularly when reporting dosages for non-thermally confined laser pulses, is highlighted by Fig. 3. When the laser pulse duration exceeds the thermal relaxation time constant, significant thermal diffusion occurs during laser delivery [34], and conventional optical dosimetry metrics lose predictive value. Radiant exposure, commonly used to report laser dosages in INS studies [36], becomes unreliable under non-thermally confined conditions because it fails to account for heat lost to thermal diffusion. This effect is exacerbated as spot diameters decrease, because the thermal time constant (Eq. 1) decreases quadratically with the spot radius. Power provides a more relevant metric for correlating laser dosages with response, but it fails to account for material properties that influence thermal dynamics during stimulation. Converting to temperature yields the most relevant metric for relating neuronal responses to photothermal laser dosages across non-thermally confined laser pulse durations. However, even the peak temperature alone cannot distinguish between a brief thermal transient and sustained heating of the same magnitude, both of which influence neuronal calcium responses. The same logic applies to reducing the thermal dynamics into a single scalar value. The optocapacitive effect, for instance, has been hypothesized to depend on the rate of temperature change, *dT/dt* [17,18]. Although the 0.5 ms pulse duration produced the largest *dT/dt* in this study (Fig. 3(D), (H)), it did not consistently elicit phasic spiking activity, suggesting that the capacitive current generated by a rapid thermal transient alone may be insufficient to initiate transmembrane calcium movement. This highlights an important finding of our work that the mechanisms underlying neuronal responses to INS are multifaceted, and our data indicate that the temporal dynamics of the thermal gradient play a central role in determining which mechanisms are recruited.

The goal of this study was to link the spatial and temporal thermal dynamics to neuronal responses. The data presented in this study provide evidence for pulse-width dependent mechanisms related to calcium signaling. We selected calcium as the response indicator because it offers insight into electrical activity and serves as a generalized secondary messenger involved in numerous cellular processes [35], such as apoptosis, long-term potentiation, and depression [42]. However, using calcium as a marker has limitations for interpreting spiking activity. A spike may reflect action potential–related activity, as seen in the prestimulation period in our data, but it also occurs under calcium-free conditions, where spiking activity would not indicate calcium influx via activation of voltage-gated calcium channels. Additionally, this method is unlikely to detect specific mechanisms demonstrated in prior work, such as effects on voltage-gated potassium channels [43] or displacement currents [17]. However, because INS is known to alter both calcium and electrophysiologic activity, it serves as a good marker for monitoring physiological responses to thermal gradients.

While there are limited studies on the effects of INS on cortical neurons *in vitro* [26,44,45], mechanistic work has identified specific calcium signaling pathways activated by infrared heating in other *in vitro* cellular models that provide a framework for interpreting the bimodal response observed in this study. All pulse durations tested in our study elicited a basal increase in intracellular calcium, both in the presence and absence of extracellular calcium, suggesting involvement of intracellular calcium release similar to previous observations in NG108 neuroglioma cells [25], HT22 and U87 neuronal cell lines [46], and cortical astrocytes [13]. Prior work suggests that IR-mediated heating affects membrane fluidity, leading to PIP_2_ hydrolysis and subsequent generation of IP_3_, which activates IP_3_R to release calcium from intracellular stores [13]. This mechanism is a plausible explanation for the intracellular component of the calcium signal observed in our study. Our data indicate that this mechanism depends on both the magnitude and duration of the thermal gradient, as the 0.5 ms pulse required the highest temperature to elicit the response phenotype. However, the calcium signals in prior studies that implicate intracellular calcium release present as a persistent elevation [25,46], which does not explain the spiking portion of the calcium signal observed under calcium-free conditions using the 50 ms pulse.

Intracellular calcium appears to partially contribute to the AC component of the calcium signal (Fig. 6). Dittami et al. suggest that mitochondrial calcium signaling is affected by infrared stimulation and demonstrated a phasic calcium response similar to those observed here [23]. Notably, their experiments employed a 400 µm optical fiber delivering laser pulses between 1.75 and 4 ms in duration, but had a thermal time constant of 67 ms, comparable to the 50 ms pulse duration used in this study. Other work in spiral ganglion neurons has proposed the involvement of TRPV4 and TRPM2 channels in intracellular calcium release, appearing as a phasic increase and subsequent decay [47]. However, in this study, the amplitude of spiking activity under calcium-free conditions was much lower than that observed in the positive control group, indicating that extracellular calcium also contributes to the spiking portions of the calcium signal.

A potential pathway for extracellular calcium entry includes heat-sensitive ion channels such as TRPV, which have been implicated in INS across several model types, including retinal and vestibular ganglion cells [20], Purkinje cells [22], cortical astrocytes [13], and dorsal root ganglion cells [21]. It remains unclear whether these channels are expressed in the neurons studied here; however, the temperatures in this study, provided by the thermal model, are sufficient to activate TRPV1–4 if present [48]. The on-gating kinetics of TRPV1 channels have been measured at approximately 6 ms, while off-gating kinetics are temperature-dependent [49]. This timing aligns closely with the 5 ms pulse duration and, as noted by Yao et al., other factors, such as membrane potential, may also influence channel open probability. Additionally, TRPV3 channels exhibit an activation half-time of roughly 29 ms, which decreases to about 8 ms after sensitization following repeated 100 ms pulses, reaching ∼53 °C per pulse [50]. These gating dynamics could explain why the 0.5 ms pulse duration failed to elicit detectable spiking responses in the control group. This theory also supports the observation that a larger spot size, with thermal confinement, provides more time for thermosensitive mechanisms to respond to the stimulus.

It is also plausible that there is some degree of alteration to neuronal excitability, leading to extracellular ion entry mediated through synaptic transmission [25–27], directly allowing calcium into the cell or triggering voltage-gated calcium channels via depolarizing ionic currents. This would be an unlikely mechanism for our experiments using the 15 µm diameter spot targeting the soma. It is a potential confounding factor when comparing the larger 400 µm-diameter spot, which stimulates the soma, dendritic processes, and other neurons, with the smaller 15 µm-diameter spot. However, as we have demonstrated in prior work, the beam profile of the 400 µm diameter spot is Gaussian, leading to a nonlinear optical and therefore thermal dosage across the irradiated zone [36], meaning that the effective stimulated area is smaller than the total beam diameter, limiting the influence of the thermal stimulus on surrounding neurons.

Cellular damage is a temperature-time dependent process described by the Arrhenius damage integral [51,52]. Our data are consistent with this principle, as the 50 ms pulse duration required the lowest temperature to elicit PI uptake. The damage threshold of spiral ganglion neurons *in vitro* was approximately 60 °C for 0.2–5 ms pulses delivered through a 200 µm fiber [53]. This is most similar to the temperature rise observed in the damage group for the 50 ms pulse duration in our study. The similarity in thresholds but the discrepancy in pulse durations may be explained by differences in the spatial distribution of the laser and its relationship to thermal confinement. The change in PI fluorescence was smaller than in our previous work, where ablated cells showed an average 8x increase from baseline [36]. The discrepancy could be explained by transient nanoporation, in which the dye enters the cell in small quantities without full membrane breakdown. The spiking observed in the damaged group for the 0.5 ms pulse duration, which was statistically elevated relative to the unreactive and reactive groups (Fig. 5(B)), indicates that calcium transiently crossed the membrane, possibly through a nanopore. Beier et al. showed that a 3.5 ms, 4.5 mJ pulse raised the temperature to ∼45 °C and maintained an elevated temperature of ∼10 °C for at least 20 ms, sufficient to induce nanopore formation [54]. Additional work is needed to validate this hypothesis and assess the potential effects of sub-necrotic damage.

## 5. Conclusion

The data presented in this study demonstrate that the temporal component of the thermal gradient influences neuronal responses. This finding indicates that mechanisms recruited by INS are at least partially, if not wholly, dependent on the temperature-time processes involved when delivering infrared light. Practically, this finding underscores the need to account for the spatiotemporal thermal gradient when designing systems for INS applications, as it determines which mechanisms are activated by the temporal dynamics of the thermal stimulus. This consideration may have further implications *in vivo*, where INS affects multiple neural cell types [10,14] and even different neuronal phenotypes [12]. A key advantage of INS is its enhanced spatial precision compared to electrical stimulation [2]. The size of the neural target ultimately dictates the dimensions of the optrode, which in turn defines the spatial characteristics of the thermal gradient. However, setting the spatial parameters of the device directly influences the temporal parameter space in which stimulation can be applied without causing thermal buildup [37]. The spatial distribution of the thermal gradient inherently affects the temporal component, inadvertently activating heat-sensitive mechanisms that may be channel-driven or arise from inherent thermal effects on the membrane. The tradeoff between spatial specificity and thermal relaxation has important implications for both the mechanisms activated by the thermal stimulus and overall safety considerations. Researchers designing IR-based neuromodulation technologies must consider the spatiotemporal distribution of the photothermal gradient relative to the specific effects they intend to elicit.

## Supplemental information

Supplement 1 Supplemental Information https://doi.org/10.6084/m9.figshare.33059141

Visualization 1 Change in laser pilot light intensity during a 5 ms, 51 mW laser pulse. https://doi.org/10.6084/m9.figshare.32411334

Visualization 2 Change in laser pilot light intensity during a 50 ms, 35 mW laser pulse. https://doi.org/10.6084/m9.figshare.32411331

Visualization 3 Representative neuronal reaction to 5 ms, 35.1 mW laser pulses applied at 1 Hz for 1 minute. https://doi.org/10.6084/m9.figshare.32411337

Visualization 4 Representative neuronal reaction to 50 ms, 19.1 mW laser pulses applied at 1 Hz for 1 minute. https://doi.org/10.6084/m9.figshare.32411328

Visualization 5 Representative neuronal reaction to 0.5 ms, 50.5 mW laser pulses applied at 1 Hz for 1 minute. https://doi.org/10.6084/m9.figshare.32411340

## Data Availability

Data underlying the results presented in this paper are not publicly available at this time, but may be obtained from the authors upon reasonable request.

## Supplemental document

See Supplement 1 for supporting content.
