# Supplementary material for: Cortical neuron calcium response to infrared neural stimulation depends on the spatiotemporal thermal gradient
Source: Biomed Opt Express. 2026 Jul 29;17(8):4474–90. doi: 10.1364/BOE.603356 (PMC13481085; doi:10.1364/BOE.603356)
Supplement: Supplementary file 1 [file boe-17-8-4474-s001.pdf]

Supplemental Document

Biomedical Optics EXPRESS

# Cortical neuron calcium response to infrared neural stimulation depends on the spatiotemporal thermal gradient: supplement

**JACOB HARDENBURGER,[1,2] GEORGE GROW,[1,2] MONA GERGES,[1,2] JOEL BIXLER,[3] CHAD OIAN,[3] BRYAN MILLIS,[1,2] CHRISTOPHER VALDEZ,[4] E. DUCO JANSEN,[1,2,5] AND ANITA MAHADEVAN-JANSEN[1,2,5,*]**

[1] *Vanderbilt Biophotonics Center, Vanderbilt University, Nashville, TN, USA*
[2] *Department of Biomedical Engineering, Vanderbilt University, Nashville, TN, USA*
[3] *Air Force Research Laboratory, Bioeffects Division, JBSA Fort Sam Houston, TX, USA*
[4] *UT Health San Antonio, San Antonio, TX, USA*
[5] *Department of Neurological Surgery, Vanderbilt University Medical Center, Nashville, TN, USA*
[*] *anita.mahadevan-jansen@Vanderbilt.Edu*

This supplement published with Optica Publishing Group on 29 July 2026 by The Authors under the terms of the Creative Commons Attribution 4.0 License in the format provided by the authors and unedited. Further distribution of this work must maintain attribution to the author(s) and the published article's title, journal citation, and DOI.

Supplement DOI: https://doi.org/10.6084/m9.figshare.33059141

Parent Article DOI: https://doi.org/10.1364/BOE.603356

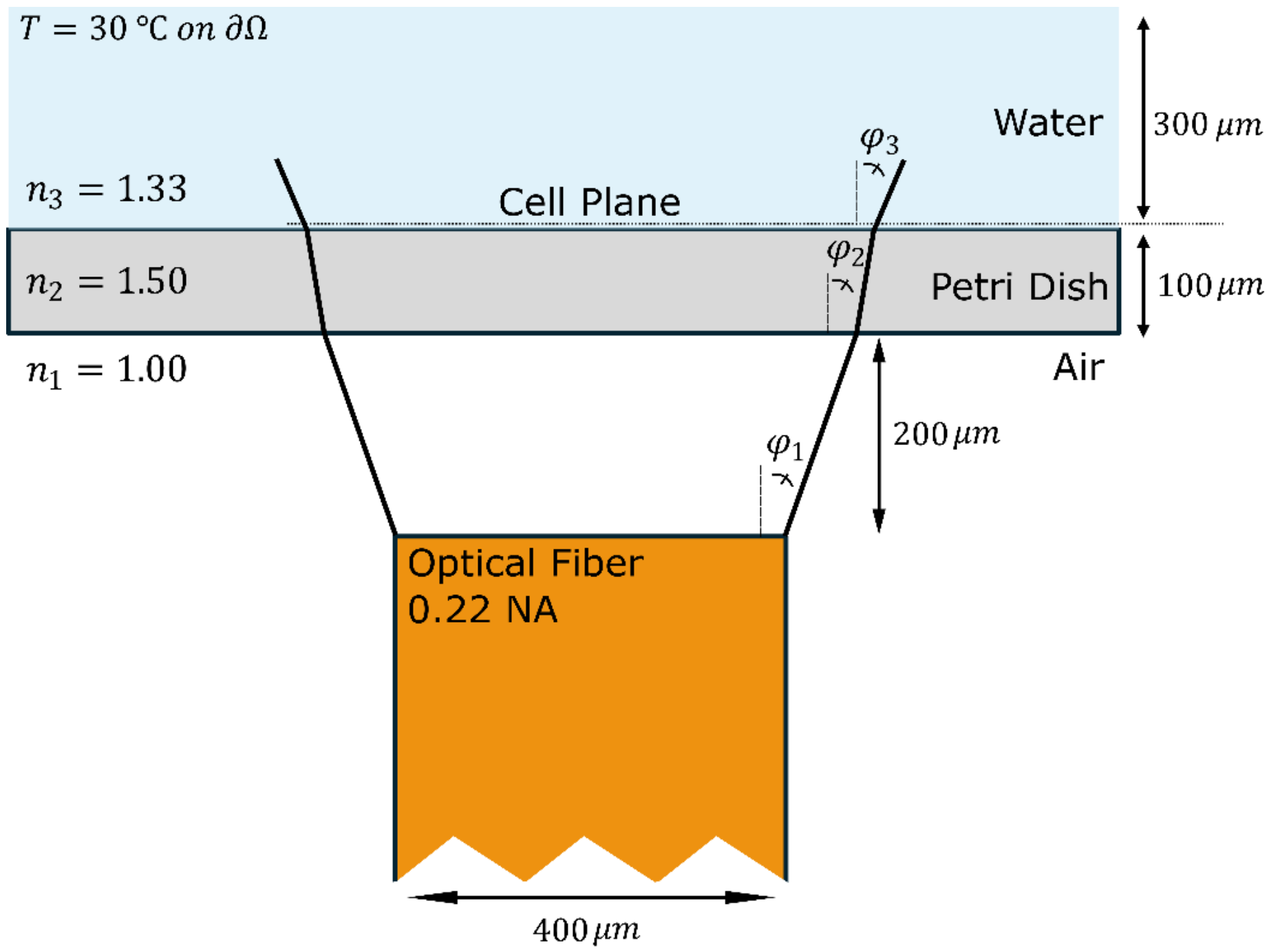


*Figure S1: Fiber irradiation geometry for experiments and thermal model.*

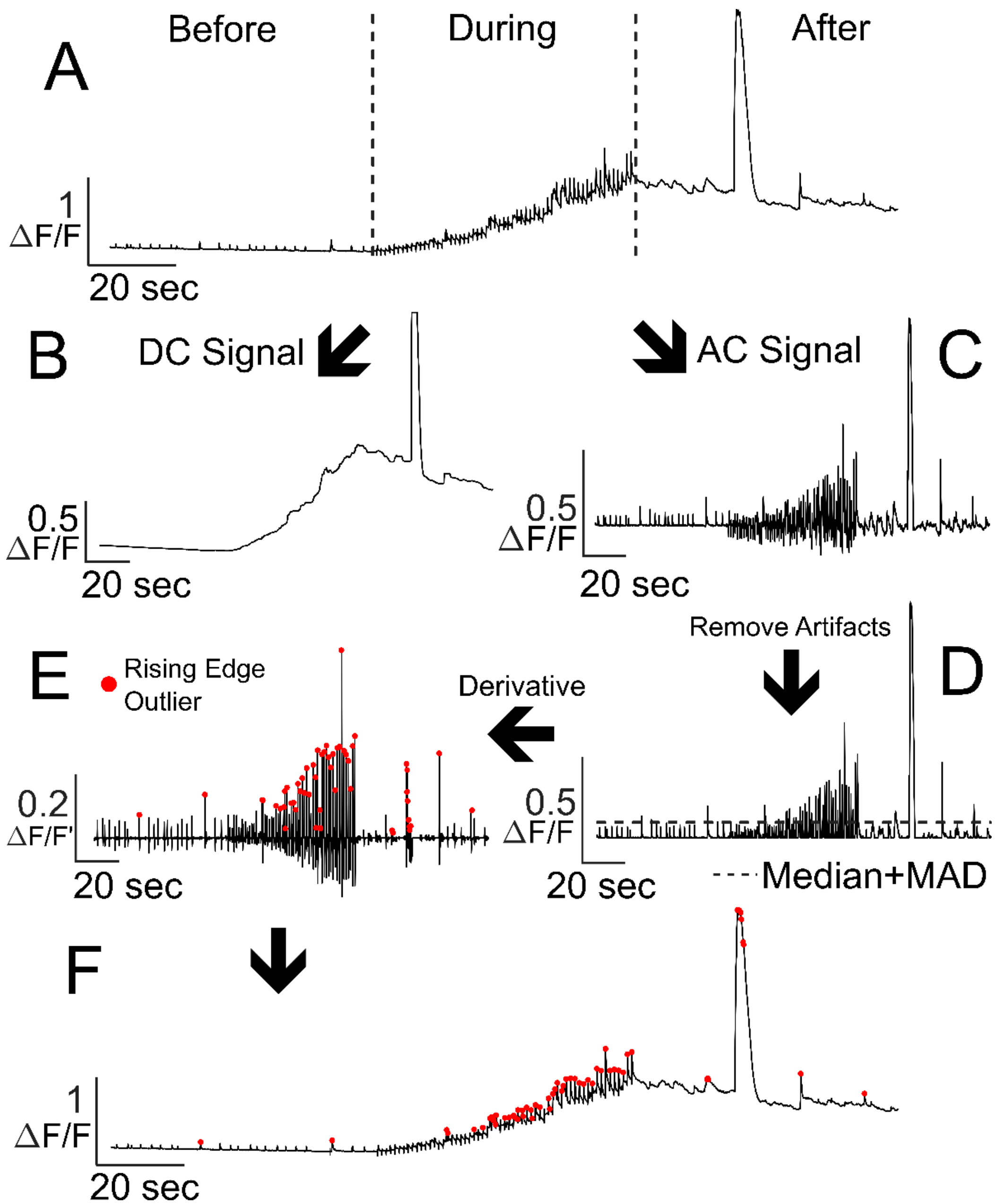


*Figure S2: A) Baseline-normalized somatic calcium signal. B) DC component of the calcium signal calculated using a moving median filter. C) AC component of the signal obtained by subtracting the trace in panel B from the trace in panel A. D) Negative part of the signal set to zero to eliminate thermal lensing artifacts. Spike threshold determined by the median plus the mean absolute deviation (MAD) of the signal. E) Derivative of the signal in panel D used to detect rising edge outliers, marked with a red dot. F) Spiking activity identified on the original signal shown in panel A.*

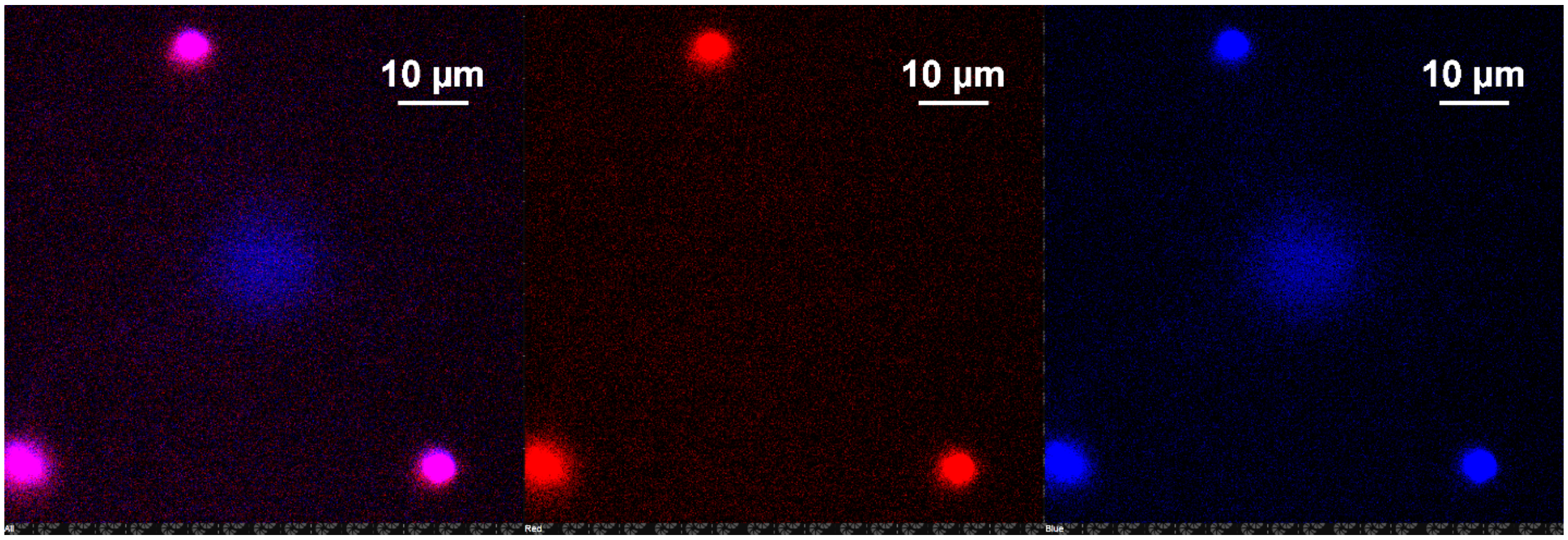


*Figure S3: Propidium Iodide signal of a damaged cell before and after irradiation protocol, merged (left), before irradiation (middle), after irradiation (right).*

Visualization 1: Change in laser pilot light intensity during a 5 ms, 51 mW laser pulse.

Visualization 2: Change in laser pilot light intensity during a 50 ms, 35 mW laser pulse.

Visualization 3: Representative neuronal reaction to 5 ms, 35.1 mW laser pulses applied at 1 Hz for 1 minute.

Visualization 4: Representative neuronal reaction to 50 ms, 19.1 mW laser pulses applied at 1 Hz for 1 minute.

Visualization 5: Representative neuronal reaction to 0.5 ms, 50.5 mW laser pulses applied at 1 Hz for 1 minute.
